# Functional and aesthetic reconstruction of a dorsal digital skin defect with a sensory neurotized DMCA III flap

**DOI:** 10.1080/23320885.2021.1942879

**Published:** 2021-07-06

**Authors:** Martin Aman, Arne Boecker, Ulrich Kneser, Leila Harhaus

**Affiliations:** Department of Hand-, Plastic and Reconstructive Surgery, Burn Center, BG Trauma Center Ludwigshafen, Department of Hand- and Plastic Surgery, University of Heidelberg, Heidelberg, Germany

**Keywords:** DMCA flap, neurorrhaphy, reconstruction, nerve, dorsal finger defect

## Abstract

The dorsal metacarpal artery (DMCA) flap is an elegant way to reconstruct tissue defects. We present a 25-year-old female patient with a dorsal injury on the fourth digit, which was reconstructed with a third webspace DMCA flap which was neurorrhaphied with a branch of the ulnar nerve, to regain sensation.

## Introduction

Covering tissue defects on the hand with local flaps is part of daily routine of a hand surgeon. Thereby, functional as well as aesthetic aspects are important considerations to be done preoperatively. Considering functional aspects, the age of the patient as well as co-morbidities in combination with the functional and aesthetic demand of the patient, the hand surgeon has a wide repertoire of reconstructive options. It ranges from skin grafting up to complex free flap coverage [1].

The neurovascular dorsal metacarpal artery flap (DMCA) for digital tissue reconstruction has been previously described for thumb reconstruction using branches of the dorsal branch of the radial nerve.

So far, no report of a dorsal ulnar branch neurotized third webspace DMCA flap is evident [2,3].

## Case report

**Figure 1.**
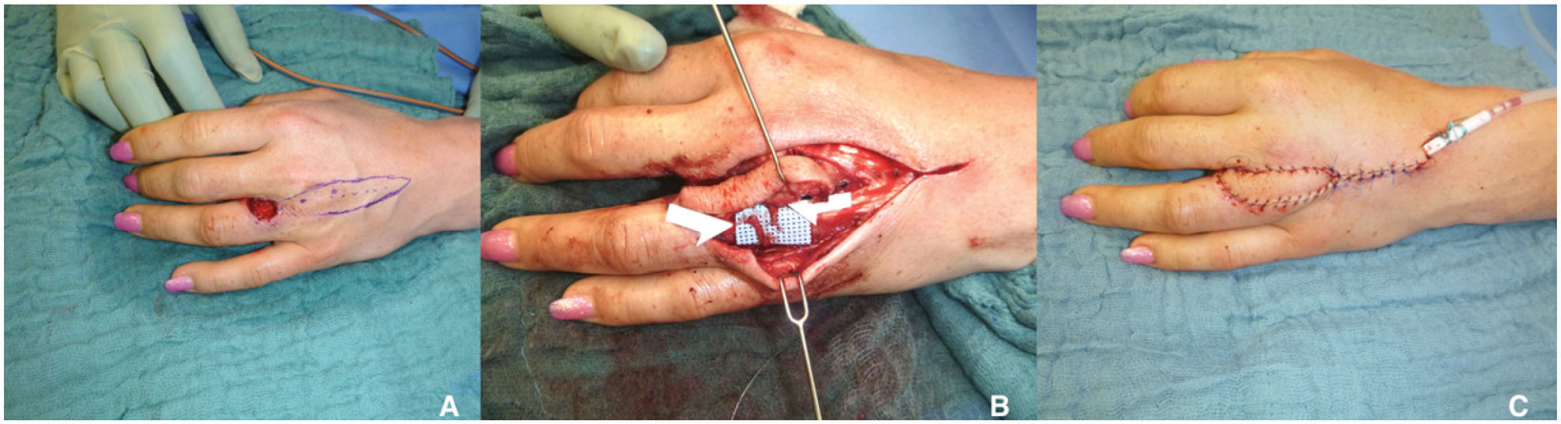
(A) Defect after excessive use of hydrochloric acid on the dorsoradial side of the fourth digit measuring about 2 × 2 cm. (B) The two marked structures are the two branches of the dorsal ulnar nerve originally innervating the DMCA flap. (C) Final result. Notice that not only a functional but also aesthetic result can be achieved with the DMCA flap.

We report a 25-year-old female patient who presented with necrosis on the proximal dorsoradial fourth digit of the left hand after excessive and repetitive use of hydrochloric acid as a wart self-treatment. After initial debridement and exploration of the seventh neurovascular bundle (radial neurovascular bundle of the fourth digit) from the dorsoradial defect zone, which was intact, a defect of 2 × 2 cm size and exposed extensor tendon apparatus with the absence of parathenon resulted. After histological and microbiological results excluded infection or malignancy the second operation was performed. For the reconstructive approach, the third webspace DMCA flap was planned (Figure 1). 

At first, the distal and proximal perforator of the dorsal metacarpal artery III were identified and evaluated for reconstruction. The proximal perforator (Quaba perforator) was favorized as the distal was too close to the zone of injury and the soft tissue showed signs of inflammation.

After preparation of the pedicle, the flap was completely raised. The branch of the dorsal ulnar nerve innervating the DMCA flap was identified on the proximal ulnar side of the flap and dissected. The cutaneous branch only innervated the skin area of the flap, so no donor side morbidity (potential loss of sensation in the digit) had to be expected. As 180-degree rotation of the flap into the defect was not feasible due to the nerve course, the nerve branch was cut, and the recipient nerve was ‘flipped’ on the undersurface of the flap around to reach the donor without tension. After assuring the integrity of the vascular flap supply was intact, an end-to-end neurorrhaphy with 9-0 Ethilon (Ethicon, Germany) microsutures was performed.

After the operation, no adverse events appeared and after suture removal a compression glove was suited. Our patient reported regaining sensation with a two-point discrimination of 10** **mm and 7/10 in the Ten test in an eight-month follow-up and showed a full range of motion.

## Discussion

Modern hand surgery provides multiple options to cover digital tissue defects. Although palmar defects are often repaired using neurotized local flaps, dorsal defects are usually considered as ‘less important’ and therefore not reconstructed with neurotized flaps.

As the loss of sensation in parts of the hand or digits can be very distracting for patients, we decided to do a DMCA flap for one-stage reconstruction and to innervate the flap with the original branch of the dorsal ulnar nerve. Initial cutting was necessary as the flap could not be rotated 180-degrees due to anatomy of the branch entering from the proximal ulnar side of the DMCA flap. By using the terminal branches of the superficial radial or ulnar nerve, a loss of sensation in the finger can be prevented by leaving the neurovascular bundle intact.

From our experience, the DMCA flap is an excellent method to reconstruct defects on the hand [4]. The flap applies evenly and provides ideal skin texture and color for hand and digital defects. Recent data demonstrates this flap as a reliable option with good flap survival rates [5].

The concept of restoring digital defects with sensate flaps is not new, yet gaining more and more popularity with the evolution of microsurgery and the challenge of the surgeon to improve his results [6,7].

Other techniques, such as full thickness skin grafting are viable options for digital defects, but also come with the burden of possible shrinkage, scarring and slight differences in skin color and texture [8]. Furthermore, skin grafts usually lack sensation [9].

Improved aesthetic and functional outcome can be achieved with a dermal substitute [10]. Although dermal substitutes provide good results, they usually require two-stage surgery and also lack sensation which could only recover be dermal wound edge sprouting. This is a viable option for many patients. Hereby, multiple factors need to be considered such as the age in combination with functional and aesthetic demands.

Hereby neurorrhaphy should not only be seen as neuroma prevention but as functional benefit especially for young and high demanding patients. With many viable options, we think this flap adds another option to the repertoire of the modern hand surgeon.
